# Sources of Power

**Published:** 1874-05

**Authors:** 


### SOURCES OF POWER.

WHAT wood and water are to the steam engine, food is to the body. By the burning of the wood, water is converted into steam, and that gives power. By the consumption of food and its digestion power is given to the body to work and the brain to think As there is more heat in some woods than others, so there is more "strength" or power, or nutriment in some kinds of food than in others. If persons have to "work hard" bodily, or have to think intensely, they must eat more than if they did not labor much, or think much.

Great thinkers are great feeders, as Count Cavour, Prince Bismarck, Charles Sumner, Henry Ward Beecher and Daniel Webster. Observant men, who have had large numbers of workmen in their employ, have long since noted the fact, that the most work could be got out of those who had the best appetite.

Hence, to study easily, that is, to get lessons easily, children and college students should eat heartily, and if they will only eat at three regular times daily, and nothing between meals, of plain, nourishing food, there is very little danger of eating too much, and there is a reasonable certainty of their having good health and becoming efficient students and fine scholars. The time may come when persons will order their food with a view to the amount and character of the labor to be performed.

